# The Evolution of Sensory Placodes

**DOI:** 10.1100/tsw.2006.314

**Published:** 2006-04-04

**Authors:** Francoise Mazet

**Affiliations:** School of Biological Sciences, AMS Building, Whiteknights, PO Box 228, Reading, RG6 6AJ, UK

**Keywords:** sensory placodes, evolution, vertebrates, urochordates, Eya, Pitx, Six

## Abstract

The vertebrate cranial sensory placodes are ectodermal embryonic patches that give rise to sensory receptor cells of the peripheral paired sense organs and to neurons in the cranial sensory ganglia. Their differentiation and the genetic pathways that underlay their development are now well understood. Their evolutionary history, however, has remained obscure. Recent molecular work, performed on close relatives of the vertebrates, demonstrated that some sensory placodes (namely the adenohypophysis, the olfactory, and accoustico-lateralis placodes) first evolved at the base of the chordate lineage, while others might be specific to vertebrates. Combined with morphological and cellular fate data, these results also suggest that the sensory placodes of the ancestor of all chordates differentiated into a wide range of structures, most likely to fit the lifestyle and environment of each species.

## INTRODUCTION

Cranial sensory placodes are key structures of vertebrate embryos, contributing to the differentiation of the cranial sensory organs and their associated ganglia. Not only are most sensory placodes found at the same relative location in all living vertebrates, but their genetic developmental programmes are identical in all vertebrate species leaving no doubt that the cranial sensory placodes arose early in the vertebrate lineage. In 1983, Northcutt and Gans proposed that sensory placode evolution was linked to the transition from filter feeders to active predators[1]. For many years, this remained the accepted idea in evolutionary theory, essentially supported by the absence of clear homologues of the sensory placodes in invertebrates.

However, sensory perception and sensory organs derived from ectodermal patches are common in animals, including insects, cephalopods, and even diploblasts. Recent molecular analyses combined with detailed morphological data have begun to challenge this hypothesis and have raised the question whether vertebrate placodes could be related to invertebrate structures. The attention was especially focused on other chordates, as they are more closely related to the vertebrates than any other invertebrates and, as such, share several key structures with vertebrates. Recently, two papers [33,36], describing the expression patterns in urochordates of canonical placodal markers demonstrate that the hypohyseal, olfactory, and acoustico-lateralis placodes are of ancient origin. In this review, I will discuss these findings and how they change our view of the evolution of the vertebrate sensory placodes.

## SENSORY PLACODES IN ANIMALS

The term "placodes" is applied to embryonic structures that can be detected first as a localised thickening of the epithelium. In vertebrates, they participate in the formation of a diverse range of structures, namely feather buds, tooth germs, sensory organs, and ganglia. Placodes can also be found in insects and participate in the development of the trachea and the cordotonal organs. Placodes that contribute to the development of sensory structures are relatively common in animals, for instance, the eye-antenna imaginal disc that gives rise to optical and olfactory structures. The cephalopod eye develops from the invagination of the ectoderm. Sensory placodes may actually be as old as the diploblasts. One group of Cnidarians, the cubozoans, possesses a complex visual system (rhopalia) that resembles the vertebrate eye morphologically, and arose from the invagination of the epidermis[2,3,4]. Interestingly, cubozoans do not possess a brain. The data from cubozoans, therefore, suggest that sensory cell formation and their focus into placodes predate the development of a central processing structure and could be as ancient as the common ancestor of diploblasts and triploblasts.

In addition to these morphological and developmental data, the majority of early placodal markers described in vertebrates are members of gene families with orthologues in invertebrates such as *Drosophila,* and diploblasts, showing the genes themselves to be of ancient origin. For instance, Pax and Six gene families are key players in the development of vertebrate sensory organs and orthologues of these genes in insects and diploblasts have similar functions[5,6,7]. Therefore, it would seem that the vertebrate sensory placodes could have evolved from some homologous structures already present in a distant ancestor, rather than being created de novo in early vertebrates. Homology of invertebrate ectodermal sensory primordia and the vertebrate cranial sensory placodes has, however, always been considered as an example of parallel evolution (homoplasy). Indeed, it is dangerous to infer an evolutionary homology between two structures solely on the basis of a loose similarity of embryologic origin (a thickened patch of ectoderm) or a single gene expression pattern in two distantly related species. For instance, developmental data regarding the development of the *Drosophila* and cephalopod eye structures show a great deal of differences that hardly support a common evolutionary origin with the vertebrate eye.

The central question is how to assess evolutionary relationships of organs in vastly different animals. Evolutionary mechanisms have produced numerous morphological variants in each animal lineage and it has to be expected that most organs will not have immediately obvious counterparts in distantly related animals. Thus, molecular data have to be associated with pertinent morphological and cellular correlations in order to demonstrate homology rather than homoplasy.

## SENSORY PLACODES IN VERTEBRATES

Vertebrate cranial sensory placodes are discrete patches of ectoderm on the head of the embryo that give rise to essential elements of the paired sense organs (eyes, ears, nose, and lateral lines) and to most of the neurons in the cranial sensory ganglia.

Six placodes are found in all vertebrate embryos in a stereotypical position relative to the neural tube. The olfactory and hypophyseal placodes develop in front of the neural tube and participate in the differentiation of the olfactory apparatus and the adenohypophysis, respectively. The lens and trigeminal placodes develop on each side of the anterior brain and contribute to the formation of the eye lens and trigeminal ganglia, respectively. The otic and epibrachial placodes develop on each side of the posterior brain and participate in the differentiation of the inner ear and the epibranchial ganglia, respectively.

In addition, lateral line placodes are found in fish and some amphibians, close to the otic placodes, and lead to the development of a population of migratory sensory cells that lie along the body of the animal. Finally, the hypobranchial placodes have been recently characterised in some species of amphibians, although it is unclear whether they have any counterpart in other vertebrates[8].

Vertebrate sensory placodes can be classified into two groups: the neurogenic placodes (olfactory, otic, lateral lines, trigeminal, and epibranchial) and the non-neurogenic placodes (adeno-hypophysis and lens placodes). Despite the difference in classification, recent work demonstrates that all sensory placodes arise from a common territory, the preplacodal region, which is then subdivided to generate each type of placode[9]. This implies that the induction of placodes begins through molecular and cellular mechanisms common to all placodes. The progressive differentiation of each type of placode is then marked by the expression of different genetic cascades. Thus, the genes involved in the differentiation of the placodes can be roughly classified into generic markers (which will make any placode) and specific markers (which will give an identity to each placode).

Early molecular markers have been identified recently for all placodes, enabling a more detailed analysis of their induction and development. An extensive range of molecular markers expressed in vertebrate cranial sensory placodes has already been characterised. The results suggested that highly complicated genetic pathways were at work. Moreover, it became clear that there were radical variations between different stages of differentiation in a particular cell lineage, different types of placodes, and different expression patterns among vertebrates. For instance, in mice, *Eya1* and *Six1* are not necessary for the induction of neurogenesis in cells derived from the otic ectoderm, as mutants for these genes still express the neurogenin gene *Neurog1* that specifies the neuroblast cell lineage. However, *Eya1* and *Six1* are needed for the maintenance of neurogenesis in the same cell lineage. In contrast, *Eya1* and *Six1* are necessary for the induction of neurogenesis in cells derived from the epibranchial placodes, as the mutants for these genes do not express *Neurog2*, which is required for the determination of neuronal precursors[10,11]. More interestingly, in chickens, these epibranchial-derived cells express the homologue of *Neurog1* instead of *Neurog2*[12]. This apparent complexity, however, reflects the plasticity of the genomes of vertebrates that had been subjected to extensive duplication events. It is actually possible to identify conserved patterns of expression during the differentiation of the vertebrate sensory placodes; all use variations of a regulatory pathway involving Pax, Six, and Eya genes. Six1/2, Six4/5, and Eya genes are widely expressed from the preplacodal stage. It seems that their function is to promote some of the basic placodal properties (for instance, morphogenesis). Six3/6 and several Pax genes are expressed more locally and are probably part of the genetic cascades that give an identity to each pair of placodes. Subsets of placodes are marked by the expression of transcription factors such as FoxI (in accoustio-lateralis and epibranchial placodes)[13,14]. Finally, the cytodifferentiation of neuronal and neurosecretory cells is marked by members of the neurogenin, Islet, Id, COE, POU IV gene families (reviewed in [15,16])

Because these gene families are consistently expressed in the sensory placodes of all vertebrates, it is likely that their basic function arose before the divergence of the vertebrate lineage itself; in short, that their orthologues in invertebrates are involved in similar functions. Thus, they are the ideal candidates to track down homologous structures in other animals.

## MORPHOLOGICAL AND MOLECULAR EVIDENCE OF SENSORY PLACODES IN UROCHORDATES

The closest relatives of the vertebrates are the cephalochordates and urochordates (Fig. 1), and consequently, most evolutionary theories regarding vertebrates have focused on these animals for decades. As a result, a wealth of morphological data, cell fate experiments, and large-scale genomic projects have allowed scientists to decipher some elusive evolutionary sequences. Morphological analyses in invertebrate chordates fuelled the argument by highlighting the strong similarities of some invertebrate structures with vertebrate placodes[17,18,19]. They were followed by the characterisation of the expression of a few conserved placodal markers[20,21,22,23]. Recently, two papers[33,36], investigated the evolution of the vertebrate sensory placodes by looking at a range of generic and specific markers in the urochordates *Ciona intestinalis* and *Oikopleura dioica*.

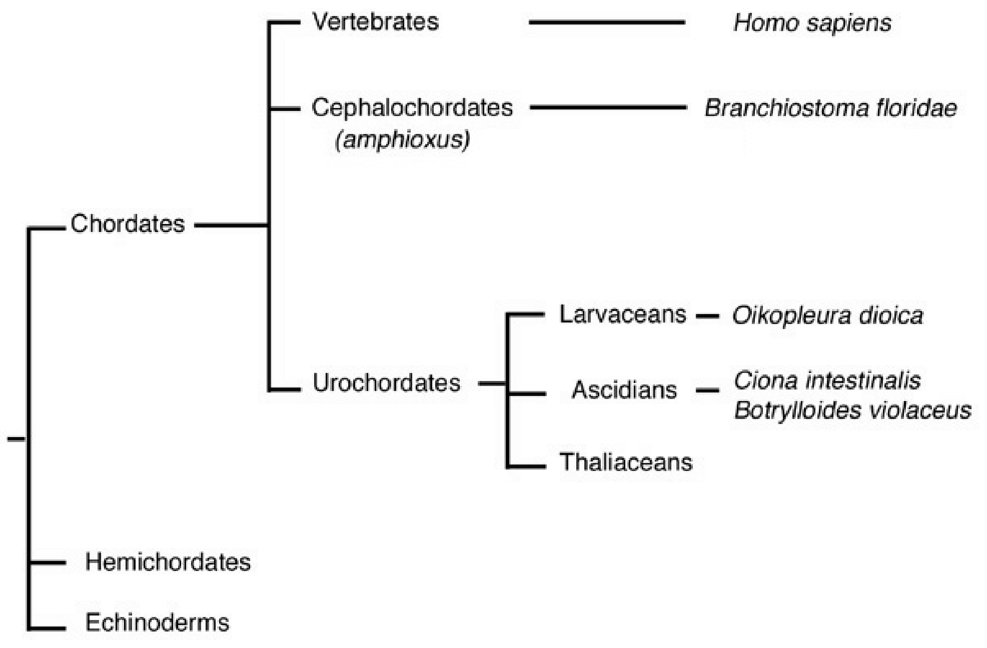

**FIGURE 1**. Phylogenetic tree of the different phyla of the Deuterostomia. The chordate phylum is divided into three subphyla: urochordates, cephalochordates, and vertebrates. Examples of species relevant for this review are listed on the right.

Several sensory organs are easily detectable in the developing *Ciona*: a light-sensitive cell (the eye spot) and a gravity-detecting cell called otolith, both located inside the cerebral vesicle and developing from the neural tube[24,25]. In larvae, sensory cells are also widely distributed over the epidermis[26,27]. *Ciona* also possess chemosensory organs called the palps, located at the very front of the larval body and containing primary sensory neurons[28]. During development, several patches of thickened ectoderm appear and invaginate in the body of the embryo: the oral primordium (also called stomodeum) that forms and invaginates in front of the brain and a pair of patches located on the dorsal-posterior ectoderm, adjacent to the posterior brain, called the atrial primordia. At the larval stage, the oral primordium fuses with the endoderm and part of the cerebral vesicle and gives rise to various organs like the ciliated funnel (suggested to be homologous to the vertebrate pituitary gland), the endostyle (the precursor of the vertebrate thyroid), and the gill slits. The two atrial invaginations fuse during metamorphosis to form the atrial (or exhalent) siphon (Fig. 2A). Despite the morphological similarities with vertebrate placodal development, the siphons have not been widely considered as candidates for sensory organs as their adult functions are not obviously associated with any sensory detection. However, ciliated and neuronal cells have been characterised in both siphons[29,30,31,32]. Recently, the expression of many placodal markers, generic or specific, has been examined in *Ciona* and it was shown that many of them are expressed in the anterior ectoderm, including the oral primordium and/or in the atrial primordium[33,34]. These ectodermal domains are marked by at least one member of the Pax, Six, and Eya gene families, indicating that this pathway operates in these ascidian structures as it does in vertebrate placodes. Additional genes orthologous to those expressed in subsets of vertebrate placodes also mark the siphon primordia. *Ci-Pitx* is expressed in the oral siphon primordium[21,22] and is expressed again later in development in the ciliated funnel historically proposed as an adenohypohyseal homologue[18]. The orthologue of the conserved chemosensory marker COE is expressed in two ectodermal cells, located directly in front of the oral primordium and known to participate in the development of the palps. Similarly, two *Ci-FoxI* genes are expressed in the atrial siphon primordia, historically proposed as otic placodes homologues. Evidence of neurogenesis in the atrial domains is delayed until metamorphosis, with the expression of *Ci-ngn* and *Ci-phox2*[17,33,35].

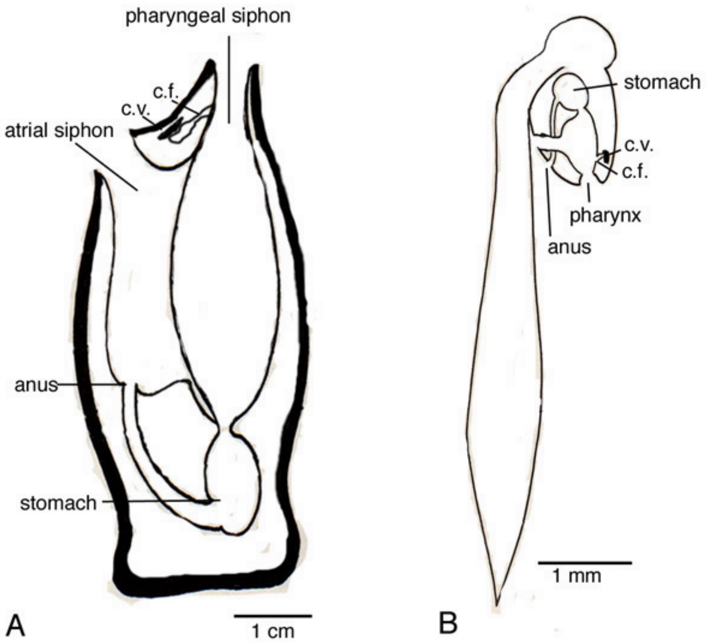

**FIGURE 2**. (A) Schematic representation of a *C. intestinalis* adult. The chordate body plan of the embryo and larvae has been drastically reorganised during metamorphosis. Sea water enters the pharyngeal siphon (which develops from the oral primordium) and exits via the atrial siphon (which develops from the atrial primordia). The ciliated funnel (c.f.) is connected to the pharyngeal siphon and to the neural gland that lay next to the cerebral vesicle (c.v.). (B) Schematic representation of an *O. dioica* adult. The adults keep their larval morphology at the adult stage and possess a tail.

Thus, these ectodermal structures, already compared with vertebrate placodes on the basis of morphological and cellular similarities, are also marked by genes involved in regulating vertebrate placode development. Therefore, the molecular and morphological data agree in identifying these areas of ascidian ectoderm as the likely homologues of vertebrate cranial sensory placodes.

The expression of placodal markers was also investigated in another urochordate *O. dioica*[36]. *Oikopleura* is distantly related to *Ciona*, but has a different developmental programme, not undergoing metamorphosis, and, notably not possessing an atrial siphon (Fig. 2B). *Oikopleura* has two obvious sensory organs: a single statolith inside the cerebral vesicle[37,38] and an ectodermal thickening close to the mouth that differentiates into the “ventral organ”, which is referred to as a possible olfactory organ[39]. The stomodeum invaginates to form the pharynx and the ciliary funnel, supposed to be homologous to both adeno- and neurohypophyses[40,41].

Bassham and Postlethwait[36] demonstrated that *Pitx* and *Six3/6* orthologues are expressed in the stomodeum and part of the pharynx that will form the ciliary funnel, a result consistent with a homology of the funnel with the adenohypophysis. The ventral organs that develop as a pair of ectodermal thickenings, close to the stomodeum, express *Eya*, *Pitx,* and *Six1/2* orthologues. The cells differentiate as primary neurons, resembling vertebrate olfactory receptor cells. However, there is no obvious relationship between the ventral organ primordia and the olfactory placodes. Rather, they are broadly located at the same topographical location as the palps cells in *Ciona*.

Several types of ciliated sensory cells can also be found in the mouth (circumoral ciliated receptors) and in the Langerhans organs that develop from two bilateral patches of ectoderm located on each side of the posterior brain. Despite morphological homologies with the vertebrate placodes, both the Langerhans and the circumoral organs had been thought to evolve independently. However, Bassham and Postlethwait[36] described the expressions of *Eya*, *Pitx*, and several Six orthologues, and concluded that some degree of homology can be found between the vertebrate sensory placodes and some ectodermal domains of *Oikopleura* embryos.

The Langerhans organs might be the homologues of the otic placodes on the basis of the expression of *Eya* and *Six3/6* orthologues and of their posterior location, adjacent to the homologue of the hindbrain. It has to be noted, however, that *Six3/6* orthologues are not expressed in the vertebrate otic or lateral line placodes, nor are they in the *Ciona* atrial placodes. Therefore, this provides only rather weak support for a homology with vertebrate accoustico-lateralis placodes. More importantly, however, the sensory neurons that develop in the Langerhans organs are secondary neurons, as in the vertebrate otic and lateral line apparatus. The circumoral mechanosensory organs are also secondary receptors. The ectoderm around the mouth expresses *Eya*, *Pitx*, and *Six1/2* orthologues, although these organs have no clear vertebrate homologues.

One has to note that the molecular and morphological evidence from *Oikopleura* are still rather weak, allowing for the possibility that we are actually looking at an example of independent evolution. Critical features are present, however: The ectodermal patches expressing the placodal genes *Eya, Pitx, Six1/2,* and *Six3/6* have similar locations, relative to the neural tube, i.e., in front of the brain and on each side of the posterior part of the brain, and the cells will develop as sensory ciliated cells.

Both papers[33,36] thus provide support for the theory that the origin of the sensory placodes precedes the split between urochordates and vertebrates. A definitive conclusion will, however, require the demonstration that these markers do indeed play a role in the differentiation of subsequent sensory cell lineages. In addition, more markers will have to be characterised to understand the molecular context of the evolution of the sensory placodes in chordates.

## ONE OR MANY ANCESTRAL PLACODES?

Not only do Mazet and colleagues[33], and Bassham and Postlethwait[36], agree that homologous structures of the vertebrate sensory placodes were characterised in urochordates, but also that some degree of diversity of the placodes predates the evolutionary origin of the vertebrates. At least three types of placodes seem to have evolved in an early chordate ancestor: the adenohypophysis, olfactory, and accoustico-lateralis placodes (Fig. 3). To this date, there are no data, molecular or morphological, to suggest that the trigeminal and eye lens placodes are present in urochordates. Results from *Ciona* give a contrasting conclusion regarding the origin of the vertebrate epibranchial placodes and ganglia: Most genes that mark this set of posterior placodes are also expressed in the otic placodes with the exception of *Phox2b*. Its *Ciona* orthologue, *Ci-Phox2*, is expressed in the invaginating atrial siphon during metamorphosis, suggesting that all three vertebrate posterior placodes have originated from a single protoplacode. The morphological data, however, do not support this hypothesis, but rather point to an ancestral role of this gene for the differentiation of ciliated sensory cells.

The presence in vertebrates of a preplacodal domain encompassing all presumptive placodes and marked by the widespread early expression of some genes like *Eya*, *Six1/2*, and *Six4/5* points at a single protoplacode in early vertebrates[42]. This hypothesis is somewhat supported by the work from Burighel et al.[29] that describes the presence of a second array of ciliated cells within the pharyngeal siphon of most urochordates. Moreover, in at least two species, *Botryllus schlosseri* and *Botrylloides violaceus*, these cells are secondary sensory cells, the same type as the neurons found in the vertebrate accoustico-lateralis apparatus. The authors conclude that the array, called the coronal organ, might be homologous to the otic/lateral line placodes. Homologues of anterior and posterior vertebrate placodes would then be located in a single anterior patch of ectoderm and have the same embryological origin. In both *C. intestinalis* and *O. dioica*, however, the expression domains of early placodal genes are always clearly separated. It is still possible, though, that the urochordates placodal domains emerged from a single preplacodal region, the markers of which remain to be characterised. Bassham and Postlethwait[36] suggest that the panplacodal field might have disappeared. Alternatively, it is also conceivable that the preplacodal domain emerged in early vertebrates with an expansion of the expression domains of *Eya* and *Six1/2*.

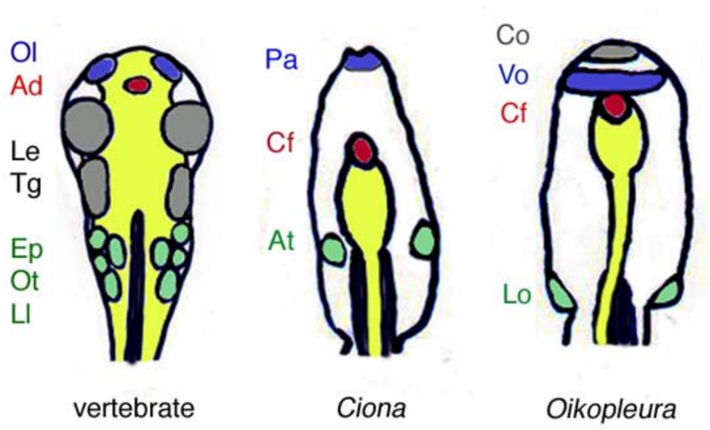

**FIGURE 3**. Comparison of vertebrate, *C. intestinalis*, and *O. dioica* placodes during development. Anterior is up for all animals. The homology between vertebrate and urochordate has been established on the basis of molecular, cellular, and morphological data. The *Ciona* palps (blue) and the *Oikopleura* ventral organ are considered homologous to the vertebrate olfactory placodes. The ciliated (or ciliary) funnel of the urochordates is homologous to the adenohypophysis, while the atrial placodes (*Ciona*) and the Langerhans organs primordia (*Oikopleura*) are homologous to the otic and, possibly, the lateral line placodes. The expression of *Ci-phox2* in the atrial primordia might suggest that the vertebrate epibranchial placodes originate from the same posterior protoplacode than otic and lateral line placodes. No evidence of homology has been found so far for vertebrate lens and trigeminal placodes. The relation of the *Oikopleura* ventral organ is unclear. The central nervous system is labelled in yellow. Placodes labelled in grey do not seem to have homologous structures in other animals. Notochord is in black. Ad: adenohypophyseal, At: atrial primordium, Cf: ciliated funnel, Co: circumoral organs, Ep: epibrnchial, Le: lens, Ll: lateral line, Lo: Langerhans organs, Ol: olfactory, Ot: otic, Pa: palps, Tg: trigeminal, Vo: ventral organs.

## AMPHIOXUS SENSORY PLACODES?

Cephalochordates represent the third group of the chordate phylum (Fig. 1). They are well known for their morphological similarities with the vertebrates and, as such, appear to be good candidates to help settle the question of the evolution of the sensory placodes. The pharynx of amphioxus, urochordates, and vertebrates are undoubtedly homologous. The atrium of amphioxus develops in larvae as an ectodermal invagination that surrounds the pharynx. In addition, Ruppert described the presence of ciliated cells and primary neurons in the atrial lining[43]. The characterisation of typical placodal markers like *Pax2/5/8*, *Sox1,2,3*, *Dach*, and *Tbx1* in embryos and larvae do not, however, suggest that placode homologues can be found in amphioxus[44,45,46,47]. The expression patterns of the canonical markers *Six1/2*, *Six3/6*, and *Eya* in amphioxus remain to be published, but preliminary reports do not support the conclusions from urochordates[48]. Amphioxus molecular data would thus appear to favour convergent evolution of the urochordate and vertebrate sensory placodes. Alternatively, cephalochordates might have lost the sensory placode(s) of their ancestors.

Another interesting aspect of the *Ciona* results is the differences of developmental stages at which some genetic cascades are activated. In vertebrates, which lack drastic metamorphosis, every developmental program is deployed during embryogenesis. In *Ciona*, however, the evidence suggests that neurogenesis in the posterior placodes is delayed until metamorphosis[33]. Interestingly, a similar delay has been observed in amphioxus for the expression of another developmental gene[49]. It is also clear that the extent of embryogenesis is shorter in invertebrate chordates than in vertebrates, suggesting that some developmental programs may start at later stages[50]. Hence, there is still the possibility that evidence of placodal domains can be found in postmetamorphic amphioxus larvae.

## CONCLUSIONS

Molecular and/or morphological evidence has recently converged to support the hypothesis that some vertebrate placodes appeared not at the basis of this lineage, but rather in a distant ancestor at the basis of the chordate lineage. Chordate sensory placodes seem to be devoted to the differentiation of ciliated sensory cells, using conserved molecular pathways, features that can be found widely in the Metazoa. It seems that evolutionary modifications rather created a wealth of morphologies supporting the sensory epithelia or subspecialising it for the detection of more types of signals[51]. Hence, the data from urochordates do not completely solve the mystery of the evolution of the vertebrate sensory placodes. It is likely that many genes have been recruited in early vertebrates to give rise to the paired sensory organs seen in modern vertebrates. The evidence also hints at a progressive evolution of the different placodes, and that, possibly, some placodes (i.e., trigeminal, lens, and epibrachial) are specific to the vertebrate lineage, but they fail to give a definitive clue regarding the sequence of events.

Finally, the likelihood of parallel evolution of the urochordate and vertebrate placodes cannot be definitively refuted. These data rather emphasise a major problem when dealing with the evolution of the vertebrates: To date, there is no plausible fossil record of an ancestral vertebrate or urochordate, let alone an ancestral basal chordate. Thus, evolutionary theories have to be deduced from data provided by animals that diverged from each other at least 550 million years ago, a period of time long enough to accumulate genetic and phenotypic modifications that suited their own evolutionary history. The classical view of chordate evolution is a linear progression with an accumulation of novelties at each step. However, the recent data regarding placode evolution, as well as other reports about the evolution of the central nervous system[20,52], hint at the fact that each lineage more likely developed or independently lost different parts the body of a common ancestor, depending on its own needs.

## ACKNOWLEDGEMENTS

I would like to thank J. Millard, S. Humphries, and S. Tops for comments on this manuscript.
